# Supplementary material for: Investigating latent syphilis in HIV treatment-experienced Ethiopians and response to therapy
Source: PLoS One. 2022 Jul 12;17(7):e0270878. doi: 10.1371/journal.pone.0270878 (PMC9275702; doi:10.1371/journal.pone.0270878)
Supplement: Attachment — Submitted filename: Response to the reviewers 5-15-2022.docx [file pone.0270878.s002.docx]

**Date: 5/15/2022**

**To: Dr Giordano Madeddu**

**Academic Editor**

**PLOS ONE**

**Subject:** Response to questions raised by reviewer #2 on the manuscript entitled "Investigating latent syphilis in HIV-treatment experienced Ethiopians and response to therapy." PONE-D-21-26734

Dear editor:

Greetings,

We want to thank you for the valuable comments the reviewers gave us to improve the quality of our manuscript. As a result, we did several editorial works to ensure the clarity and continuity of our work. The revised manuscript is attached for your comments. We have also made the raw data available. Below you will find our response to the questions raised by the second reviewer.

Response to the questions raised by reviewer 2:

**Question #1**: The authors wrote, "The indications were RPR titer > 1:32, peripheral blood CD4 count < 350 cells/mm^3^ if the HIV RNA level is above 200 copies/mL," and "We included the HIV RNA level criterion to exclude immunological nonresponders (a category of HIV-infected patients on cART who have suppressed HIV viremia but a suboptimal increase in their CD4 cell count)". This part is unclear. Neurosyphilis could also be present in people with more than 350 CD4+ cells/mm3. Please comment.

**Authors' response:** We accept the section on "diagnosis of neurosyphilis" was not explicit. Therefore, it is written again (refer to page 7). The indications for CSF analysis in our study were a/ serum RPR titer ≥ 1:32 b/peripheral blood CD4 count ≤ 350 cells/mm^3^, and HIV RNA level above 2.3 log/mL (or 200 copies/mL) c/serological nonresponse or treatment failure after latent syphilis therapy and d/unexplained persistent neurologic, ocular or otic symptoms or signs. CNS invasion with *Treponema pallidum* in PWH can occur at any stage of syphilis, CD4 count, and RPR titration. It may be asymptomatic or manifests with central nervous system affection symptoms. Asymptomatic neurosyphilis, however, rarely occurs in antiretroviral naïve PWH and syphilis confection with RPR titer values below 1:16 and > 350 peripheral CD4+ cells/mm^3^. Therefore, it is controversial that this group should require routine CSF evaluation. Several studies have suggested that serum RPR titers ≥ 1:32 or CD4 counts ≤ 350 in treatment naïve HIV-syphilis coinfected patients indicate a higher risk of asymptomatic infection. Thus, the current guidelines recommend evaluating the CSF for neurosyphilis based on these suggestions. The guidelines fail to address the risk of asymptomatic neurosyphilis in PWH with suppressed HIV RNA levels due to combination antiretroviral therapy (cART). In patients with CD4 counts > 350 and RPR titer ≥ 1:32, CSF evaluation is highly recommended regardless of symptoms. There is a potential to miss asymptomatic cases presenting without symptoms and an RPR titer < 1:32 unless CSF analysis is done routinely. Since we didn't perform CSF analysis in all our latent syphilis cases, there is a potential missing, even though it is rare. It is one of our study limitations (page 13, lines 270-71). We didn't expect the RPR titer to show a favorable response with either BPG or DOXY therapy when there is neurosyphilis. It was the reason for including serological nonresponders and treatment failure cases for CSF analysis to rule out neurosyphilis. We conclude that it is quite rare for neurosyphilis to occur without symptoms in PWH on suppressive cART, CD4 above 350, and latent syphilis coinfection, provided the RPR titer value is below 1:32.

**Question #2**: The authors wrote, "Neurosyphilis was diagnosed with a reactive CSF VDRL test or the presence of pleocytosis (WBC > 10 cells/mm3)". Therefore, I suppose that a patient with negative CSF VDRL but 11 WBC in the CSF was considered positive for neurosyphilis. It could represent an important bias that could cause an overrate of neurosyphilis. Please comment.

Have patients with a diagnosis of latent syphilis performed an ophthalmic evaluation? Please comment.

**Authors' response.** The diagnosis of neurosyphilis is a complex process. CSF WBC count > 10 cells/mm^3^ is often used as a diagnostic criterion for neurosyphilis in PWH. However, the specificity is lower in PWH, for there are other pleocytosis causes outside of neurosyphilis. If this criterion alone is used, it will certainly lead to an overdiagnosis of neurosyphilis, particularly in PWH without symptoms. The level of uncertainty would decrease if both criteria are used together (CSF VDRL and WBC > 10 cells/mm^3^); however, the problem is the CSF VDRL lacks sensitivity. Even if using this criterion, none of the seven CSF we analyzed supported the diagnosis of neurosyphilis, implying syphilis disease activity is reduced in the latent stage. We conclude that neurosyphilis from latent syphilis infection is rare in PWH receiving virus suppressive cART and RPR titers below 1:32.

We did ophthalmic examination in cases diagnosed with latent syphilis ( page 6, lines 121-122).
